# Coenocline Simulation of Microbiome Samples: A Biologically Mechanistic Framework for Generating Ecologically Realistic Synthetic Datasets to Support Classification Method Evaluation

**DOI:** 10.3390/pathogens15080877

**Published:** 2026-08-21

**Authors:** Cameron Hurst, Dhammika Leshan Wannigama, Eva Malacova, Pichaya Tantiyavarong, Nop Khongthon, Anita Pelecanos, Lee Jones, Robert Hurst, Gunter Hartel

**Affiliations:** 1 Department of Clinical Epidemiology, Faculty of Medicine, Thammasat University, Pathum Thani 12120, Thailand; cphurst@gmail.com (C.H.);; 2 Center of Excellence in Applied Epidemiology, Thammasat University, Pathum Thani 12120, Thailand; 3 Statistics Unit, QIMR Berghofer Medical Research Institute, Brisbane 4006, QLD, Australia; 4 Pathogen Hunter’s Research Collaborative Team, Department of Infectious Diseases and Infection Control, Yamagata Prefectural Central Hospital, Yamagata 990-2292, Japan; 5 Department of Infectious Diseases, Faculty of Medicine, Yamagata University, Yamagata 990-2292, Japan; 6 Department of Infectious Diseases and Infection Control, Yamagata Prefectural Central Hospital, Yamagata 990-2292, Japan; 7 Biofilms and Antimicrobial Resistance Consortium of ODA Receiving Countries, The University of Sheffield, Sheffield S10 2TN, UK; 8 Center of Excellence for Vectors and Vector-Borne Diseases, Faculty of Science, Mahidol University, Bangkok 10400, Thailand; 9 Graduate School, Yamagata Prefectural University of Health Sciences, Kamiyanagi, Yamagata 990-2212, Japan; 10 Griffith Institute for Biomedicine and Glycomics, Giffith University, Southport 4222, QLD, Australia

**Keywords:** coenocline simulation, microbiome classification, mechanistic ecological modeling, ecological gradients, community assembly, beta diversity

## Abstract

Machine learning and statistical classification methods are widely applied to microbiome data for diagnostic, prognostic, and phenotypic insights. However, the complex, multivariate nature of microbiome communities makes it difficult to assess the relative performance of these methods. Most comparisons rely on a small number of published datasets, without considering their underlying ecological properties or how these properties may, in turn, influence classification performance. We introduced a coenocline-based simulation framework to generate synthetic microbiome datasets that incorporate realistic ecological variation arising from species’ responses to host-associated gradients such as disease severity. To evaluate the ecological fidelity of these simulations, we compared synthetic datasets to five widely used real-world microbiome datasets: Cirrhosis, Colorectal Cancer (CRC), Type 2 Diabetes (Chinese and Women cohorts), and the Human Microbiome Project (HMP). Comparisons across α-diversity (species richness), β-diversity (species composition and turnover), and abundance distributions demonstrated that coenocline simulations closely recapitulate the key ecological structures of empirical data. Synthetic datasets exhibited similar richness and abundance patterns to disease-associated microbiomes, with realistic distributions of few dominant and many rare taxa. Moreover, community composition analyses (Bray–Curtis index) revealed that the simulated datasets captured natural levels of compositional dissimilarity among samples, spanning the same variability range observed in real data. When compared against 100 independently simulated datasets, the coenocline model consistently reproduced empirical ranges of species diversity, relative abundance, and between-group compositional differences (ANOSIM-R values), confirming the model’s robustness and reproducibility. This coenocline-based simulation framework provides a novel, flexible, and ecologically grounded approach for generating synthetic microbiome data with controlled complexity. By reproducing realistic ecological gradients and community structures, the framework supplies the controlled test beds needed for systematic future benchmarking of machine learning and statistical classification methods across diverse and biologically meaningful scenarios. In doing so, it will help bridge the gap between ecological realism and computational modeling, thereby supporting more reliable and generalizable inference from microbiome data.

## 1. Background

The microbiome encompasses a diverse array of microorganisms, comprising bacteria, viruses, and fungi, that inhabit a host organism’s interior and exterior surfaces [1]. This intricate ecosystem plays a pivotal role in influencing the susceptibility and intensity of various diseases within the host [2]. The interplay between the host and its microbiome is complex, with the composition and balance of these microorganisms contributing significantly to health and disease outcomes in the host organism [1,2]. Microbiome datasets collected by high-throughput sequencing of 16S rRNA gene amplicons, metagenomes, or metatranscriptomes represent crucial tools for investigating dysbiosis, whether the focus is on understanding human disease states through the examination of phenotype-microbiome associations or exploring ecological variation in species assemblages along environmental gradients [1,2,3].

Despite the usefulness of microbiome data for diagnosis, prognosis, and studying disease progression, classification tools based on microbiome data have yet to be routinely implemented in standard clinical care [3]. There are a number of possible explanations for this. First, the field of microbiomes is relatively new, and it takes time for clinical research involving microbiomes to filter down into widespread clinical practice. A second possible explanation is that microbiome data present several analytical challenges that are yet to be addressed [4]. One such challenge is the large number of features, represented by operational taxonomic units (OTUs), contained in microbiome datasets—a challenge seen in other fields involving high-throughput biological data. However, a particular difficulty presented by microbiome datasets is that the features are represented by abundance counts of many OTUs (e.g., species or strains), most of which are rare and therefore unlikely to be normally distributed and almost certainly not collectively multivariate normal. This may present problems for many of the multivariate statistical and machine learning methods that have proven useful in the analysis of data from other ’omics fields.

Several studies have compared the ability of various statistical and machine learning classification methods using microbiome data for clinical purposes [5,6,7,8,9]. For the most part, these studies apply classification methods to a relatively small number of existing published datasets and then compare both the training- and test-set classification accuracy for these methods. For instance, Pasolli and colleagues [9] compared the classification accuracy of random forest, support vector machine, Elastic Net, and Lasso-regularized logistic regression classification methods using shotgun metagenomic data collected from patients with liver cirrhosis [10], colorectal cancer [11], IBD [12], obesity [13], and Type 2 diabetes [12,14]. One problem with this approach is that the datasets used in these types of studies vary widely in both the diseases and populations they consider, as well as the study settings in which the data were generated; differences between these exemplar datasets are manifold and, as yet, poorly understood. As a result, we gain limited insight into the impact of various types and levels of ecological difference or the role study design plays. This, in turn, affects our understanding of how classification methods can accurately predict disease status. For instance, what impact does species (or other OTU) diversity have on the accuracy of the various classification methods? What impact does compositional difference have? There are ways in which ecological assemblages (groupings of samples within a microbiome dataset) may differ, and these types of ecological variation are likely to interact in subtle ways that are still not fully understood. In addition, how might study design impact classification accuracy? For example, how does the case-to-control ratio, level of taxonomic resolution, overall sample size, and sample split for training, validation, and testing purposes affect various methods’ classification accuracy and prediction optimism?

A better approach to gauge how well the various classification methods may perform across a range of ecological and experimental situations would be to have access to a large number of realistic microbiome datasets whose ecological and study design properties vary in a systematic way. In this study, we outline a simulation method for generating artificial microbiome datasets whose ecological and study design properties can be made to vary systematically. This simulator is intended as a necessary precursor tool that will enable the assessment of machine and statistical learning methods in their relative abilities to accurately classify microbiome samples across the range of ecological and experimental conditions observed in the literature. We also outline a framework for examining the ecological properties of microbiome datasets that can facilitate the reconciliation of statistical and machine learning classification rule accuracy with the ecological properties of these datasets observed in microbiome studies.

### 1.1. Ecological Difference

Taxonomic assemblage data are multivariate, and there are several types of ecological differences that can be used to compare samples. Indeed, it is unlikely that a single metric of ecological difference can fully capture the disparity among such multivariate samples. For convenience, we will frame our definitions at the level of species, although the principles we discuss hold for other levels of taxonomic resolution as well. A comprehensive literature exists concerning the many types of ecological properties used to describe species assemblages and how we might best differentiate between different samples (or groups of samples) occupying different habitats (or disease states). We will focus on three main aspects of ecological difference: species diversity, compositional difference, and variation in abundance.

Species diversity is often quantified as the simple count of species observed in a sample (also known as species richness or α-diversity), but other measures exist that incorporate how individual organisms are distributed among species. For example, a sample where individuals are evenly distributed among species may be considered more diverse than a sample with an equivalent number of species and individuals but where a single or small number of species dominate [15]. Many measures of species diversity exist, including species richness, Simpson’s index, and Shannon’s entropy. A limitation of species diversity measures when comparing samples (or groups of samples) is that these measures do not take the identity of species into account. Two samples may have the same level of diversity but share no species in common.

To assess how samples might share species, we need to consider sample composition; that is, what species are present in a sample. In its crudest form, compositional similarity between two samples can be measured as the percentage of species shared. However, there are many metrics of compositional (dis)similarity that also account for species abundance. Indeed, the use of various data transformations, standardizations, and compositional dissimilarity measures (which often include implicit transformations and standardizations) makes any discussion of compositional (dis)similarity a complex one.

In traditional ecological studies of macroorganism species assemblages, total abundance (the sum of all individuals observed in a sample) can be seen as a measure of sample productivity. This is a useful measure when we are considering species whose abundances are relatively close in value (e.g., trees) but may not be as useful for microorganism assemblages in which level of species abundance may differ by several orders of magnitude; a species whose abundance is numerically much higher may have less impact than abundance differences in a keystone species that happens to be much rarer. Another important question when considering microbiome data is whether absolute species counts can be seen as a reliable measure of abundance. In microbiome studies, there are technical issues around read depth that call into question how trustworthy a measure of raw abundance is. For example, the bioinformatic pipeline for both 16S and shotgun metagenomic platforms involves the conversion of raw abundances to relative abundances; each sample’s species abundances sum to 100. Indeed, it has been suggested that even these relative abundances generated from microbiome datasets should be ignored as arbitrary, and only the presence/absence of species in a sample should be considered. Microbiome data should be considered purely compositional [16]. Others maintain that even though the absolute abundances obtained from a microbiome dataset are somewhat arbitrary, the relative abundances obtained from the various technologies used to generate microbiome data can still be considered consistent [2]. Regardless, relative abundances are the data used for analysis in a large majority of studies that consider the microbiome.

At least in the application of microbiome data to detect disease states, the nature of ecological differences between samples is glossed over in a majority of studies. However, if we want to consider the ability of the various classification methods to differentiate samples from diseased and non-diseased patients, we need to understand how sensitive these methods are to the various types and levels of ecological differences contained in the data.

### 1.2. Coenocline Simulation of Species Assemblages

Coenocline models are based on the notion that variation in habitat, as expressed by one or more environmental gradients, leads to changes in the occurrence and abundance of individual species, as well as variation in species diversity and composition when several species are being considered. In the context of clinical applications examining phenotype–microbiome associations, an environmental gradient might be represented by disease severity (continuous) or disease presence/absence (categorical). These coenocline models were developed and first used to examine the ability of various unsupervised multivariate projection, or ordination, methods to recover ecological patterns in assemblages of plant species [17,18,19]. There are three main steps in the specification of a coenocline model to generate artificial species assemblage data [20]: (1) Defining a species-response function (SRF) for each species contained in an assemblage, i.e., how each species responds to changes in environmental conditions. (2) Specifying a set of sample locations along a single or set of environmental gradients. Each SRF can then be evaluated at each sampling point to yield an expected species abundance. In this respect, the SRF represents a mathematical expression of the relationship between a species’ abundance and its environment. At this stage, species abundances are deterministic; a particular species-response function evaluated repeatedly at a particular gradient location will yield the same expected abundance. (3) Designating a count sampling distribution, such as the Poisson or negative binomial distribution. This will incorporate sampling variation, or noise, into the simulated data.

A further refinement of the coenocline model is the incorporation of other species’ impact on a given species response. A physiological SRF describes how the abundance of a species is governed by a gradient in the absence of all other species, whereas an ecological response represents a species response to a gradient when the interaction with other species (e.g., symbiosis or competition) is incorporated [20]. The physiological and ecological responses have been equated to the fundamental and realized niche, respectively [21].

Earlier coenocline models, such as those proposed by Gauch and colleagues [22] and Ihm and Groenewoud [23], were based on a Gaussian SRF. The main problem with this model is the assumption of symmetry along a gradient and a particular level of SRF kurtosis. Minchin [20] proposed the generalized β function, a five-parameter model that allows much more flexibility in SRF shape, allowing variation in both skewness (including its direction) and kurtosis. A single-gradient coenocline model using the generalized β function can be defined as

(1) $$\begin{array}{r} A_{ij}=\frac{A_{0i}}{d_{i}}\left[\frac{x_{j}-m_{i}}{r_{i}}+b_{i}\right]\left[1-\left(\frac{x_{j}-m_{i}}{r_{i}}\right)+b_{i}\right] \end{array}$$

where $A_{ij}$ is the expected abundance of species i for gradient level $x_{j}$, for i=1,2,⋯,k species; and j=1,2,⋯,n samples.

The ith species has the following coenocline parameters:

- $A_{0i}$ is the modal abundance of species i along the gradient;
- $m_{i}$ is the modal coordinate;
- $r_{i}$ is the niche breadth;
- $\alpha_{i}$ and $\gamma_{i}$ are parameters that collectively control skewness and kurtosis;
- $b_{i}$ and $d_{i}$ are convenience parameters, used here to simplify the form of the model in Equation (1), and are given in Equation (2) and Equation (3), respectively.

The gradient variable is defined as follows:

$x_{j}$ is the jth value (location) along a gradient (environmental variable).

(2) $$\begin{array}{r} b_{i}=\frac{\alpha_{i}}{\alpha_{i}+\gamma_{i}} \end{array}$$

(3) $$\begin{array}{r} d_{i}=b_{i}^{\alpha_{i}}{\left(1-b_{i}\right)}^{\gamma_{i}} \end{array}$$

Using model Equation (1) to generate abundances for an individual species, the same gradient value will result in the same expected abundance; the model is deterministic. To incorporate sampling variation, we can sample from a distribution whose expectation value is obtained from Equation (1). As the coenocline model is expected to produce species counts, distributions like the Poisson or negative binomial are a logical choice. Furthermore, zero-augmented versions of these distributions can also be employed to inflate the expected number of sampling zeros often seen in ecological samples. Finally, we should note that the model presented in Equation (1) can be extended to multiple gradients, with all parameters being gradient-specific with the exception of the modal abundance, $A_{0}$.

**Figure 1:**
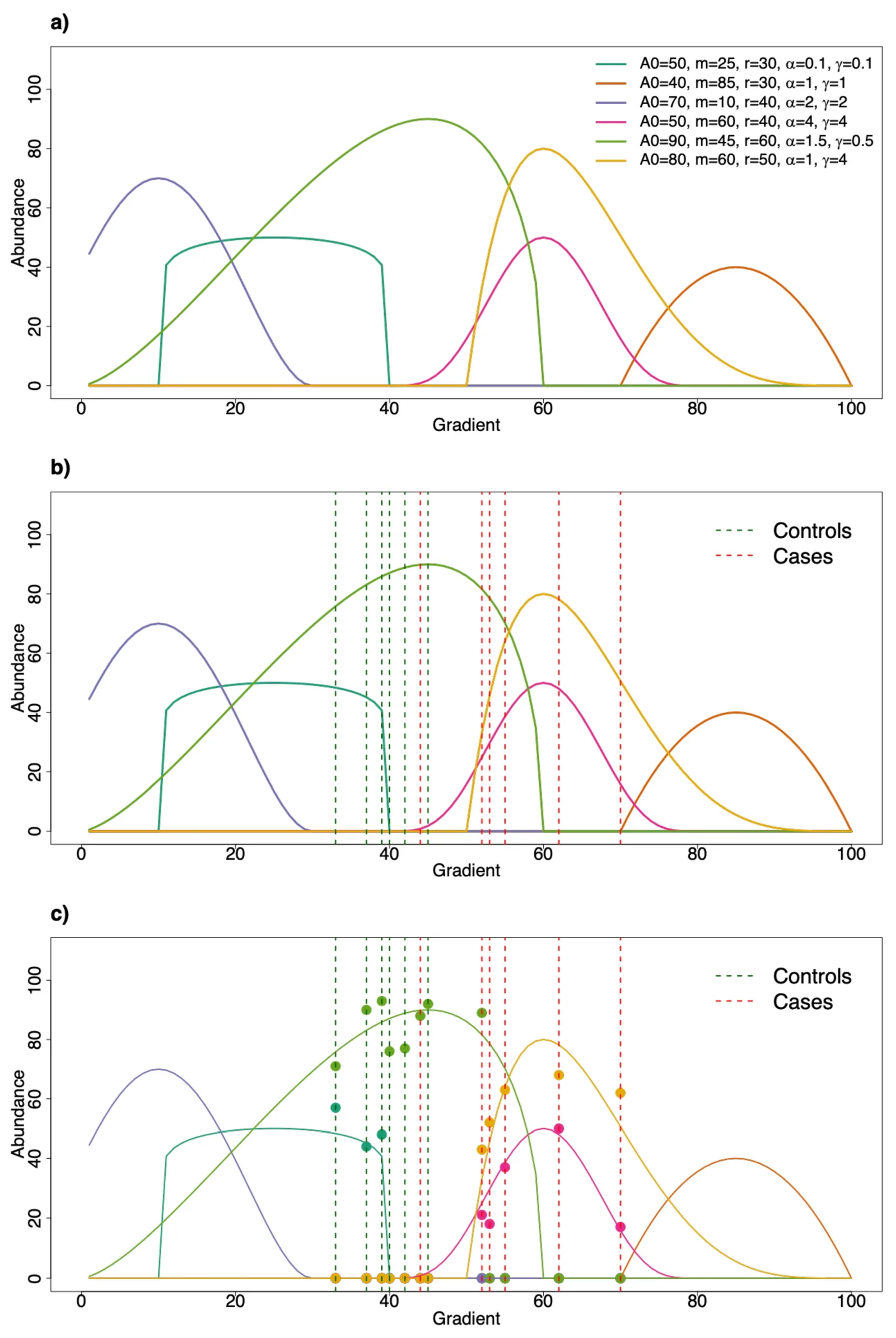
Coenoclines for six fictitious species. (**a**) We have the six species-response functions (generalized β functions) that yield the expected abundance of a species at any point on the gradient; (**b**) we then sample at certain zones of the gradient to obtain control and case samples; and (**c**) the expectations obtained from step b are subsequently used to specify a count sampling distribution (in this case, a zero-inflated negative binomial distribution) from which we obtain an artificial species abundance.

In Figure 1, we present the assemblage of six fictitious species as specified in Minchin’s original description of the generalized β coenocline model [20]. In Figure 1a, we demonstrate the first step in coenocline simulation by specifying the species-response functions (SRFs) for six species, which yielded the expected abundance of a species at any point on the gradient. In Figure 1b, we demonstrate the second step by sampling certain zones of a single environmental gradient to obtain control and case samples. Finally, in Figure 1c, the expectations obtained from Figure 1b were used to specify a sampling distribution (in this case, a zero-inflated negative binomial distribution) from which we obtained synthetic species abundances.

## 2. Methods

### 2.1. Specification of Simulation Models

Specification of coenocline models involves three steps: (1) specifying the parameters for each species’ response curve; (2) sampling the environmental gradient variable(s); and (3) incorporating sampling error through the specification of a count model.

The calibration procedure is described below.

Step 1—Select initial parameter distributions. We first selected candidate distributions for the five generalized β-function parameters (modal abundance $A_{0}$, modal coordinate m, niche breadth r, and the shape parameters α and γ) by examining the marginal distributions of species richness, maximum relative abundance, and rank-abundance curves in the five real metagenomic datasets listed in Table 1. Because these real datasets are already relativized and capture only partial species-response curves, we could not formally fit the β function to individual taxa. Instead, we chose flexible distributions whose resulting synthetic communities reproduced the observed ranges of α-diversity, dominance–rarity structure, and compositional turnover.
Step 2—Fix a priori choices. Two components were fixed before any iterative tuning:

- The form of the count model (zero-inflated negative binomial with excess-zero probability π=0.2 and dispersion θ=0.125);
- The locations of the control and case sampling zones on the single disease-severity gradient ($X_{\mathrm{control}}∼N(45,15^{2})$, $X_{\mathrm{case}}∼N(55,15^{2})$).

These choices were made early so that subsequent ecological differences would arise primarily from variation in the species-response parameters rather than from changes in the gradient sampling scheme.

Step 3—Iterative refinement and acceptance criterion. Starting from the candidate distributions, we generated batches of synthetic datasets and compared them visually and quantitatively (mean sample richness, mean maximum relative abundance, and ANOSIM-R) with the five benchmark datasets. Distributions were adjusted until the cloud of 100 independently simulated datasets spanned the observed ranges of these three summary statistics. No formal numerical optimization was performed; the process remained empirically driven because of the absence of datasets that adequately sample full species-response curves.
Step 4—Independence assumption. All parameters were drawn independently. Although dependence among parameters is biologically plausible, established theory linking the β-function parameters is lacking; we therefore adopted the independence assumption as a transparent and reproducible starting point.

The final distributions used for all simulations are given in Supplementary Table S1. All simulations were generated with the R package *coenocliner* (v0.2-4) [25]; all subsequent analyses used R (v4.6.1) [26].

### 2.2. Assessing Model Validity

To assess the validity of the coenocline model, we compared our generated synthetic microbiome dataset to five well-known published microbiome datasets spanning a number of diseases and populations [10,11,12,14,24]. All of these published datasets were generated using shotgun metagenomic sequencing and are summarized in Table 1.

All studies samples, except for the Human Microbiome Project (HMP) [24], which comprises samples from multiple body sites, does not include a case–control contrast (NA = not applicable).

We then compared our synthetic dataset to the real datasets in two ways. In the first case, we randomly selected a single simulated dataset to compare to the five benchmark datasets (Table 1) in terms of various measures of within-dataset diversity, abundance, and compositional variability. We compared the distribution of these measures from a single dataset simulated using the coenocline model with those of the real datasets. To ensure that the level of resemblance of this single synthetic dataset to the benchmark microbiome datasets was not just coincidental (i.e., a single model realization), we also compared 100 synthetic datasets to the real datasets using some dataset-level measures of diversity, abundance, and composition. The within-dataset and dataset-level measures employed are provided in Table 2.

Compositional similarity was calculated using the Bray–Curtis index, and dissimilarity using the Bray–Curtis distance measure. Both were calculated using presence-absence data to give the percentages of shared and unique species, respectively.

Perusal of the measures in Table 2 demonstrates that they summarize the three different aspects of ecological difference we evaluated in the present study: diversity, abundance, and composition. First, species diversity was assessed using both species richness (also known as α-diversity), the number of species observed, and log-rank abundance, which also considers how individual microorganisms are distributed among species. Second, abundance was measured by considering both the overall average (dataset-level description) and the distribution of individual species’ maximum relative abundance (within-dataset level). Generally, we would expect only a few species to have high relative abundances, whereas the large majority of species will be quite rare. With the third and final set of measures, those concerned with compositional similarity/difference, the identity of individual species becomes important. For the within-dataset measure, we considered compositional similarity as represented by the Bray–Curtis index calculated using presence/absence-transformed data, which represents an index of the percentage of species shared between two samples. For the within-sample comparison (our single simulated dataset vs. the five published datasets), we examined the distributions of both within-group and between-group (cases vs. controls) Bray–Curtis index values, which provide a comparison of the observed levels of within- and between-group compositional variation. For the dataset-level composition (100 synthetic sets vs. 5 benchmark datasets), we used the ANOSIM-R statistic [27], which represents a relative measure of between-group and within-group (rank) dissimilarity. Generally, the greater the ANOSIM-R statistic, the higher the between-group compositional difference is relative to within-group dissimilarity. An ANOSIM-R = 0 implies that compositional dissimilarity among between-group samples is similar to compositional dissimilarity among within-group samples.

## 3. Results

We compared synthetic datasets simulated with the coenocline model to five widely used published microbiome datasets [10,11,12,14,24]. These published datasets (hereafter referred to as the benchmark datasets) include microbiome datasets from four studies that consider both cases and controls: Cirrhosis [10], Colorectal Cancer [11] (CRC), Type 2 Diabetes in the Chinese Population [12] (T2DM.C), and Type 2 Diabetes in Women [14] (T2DM.W). The microbiome dataset from the fifth study involves a single, albeit broader, population, but with microbiome samples taken from several sites on the body: the Human Microbiome Project [24] (HMP).

**Figure 2:**
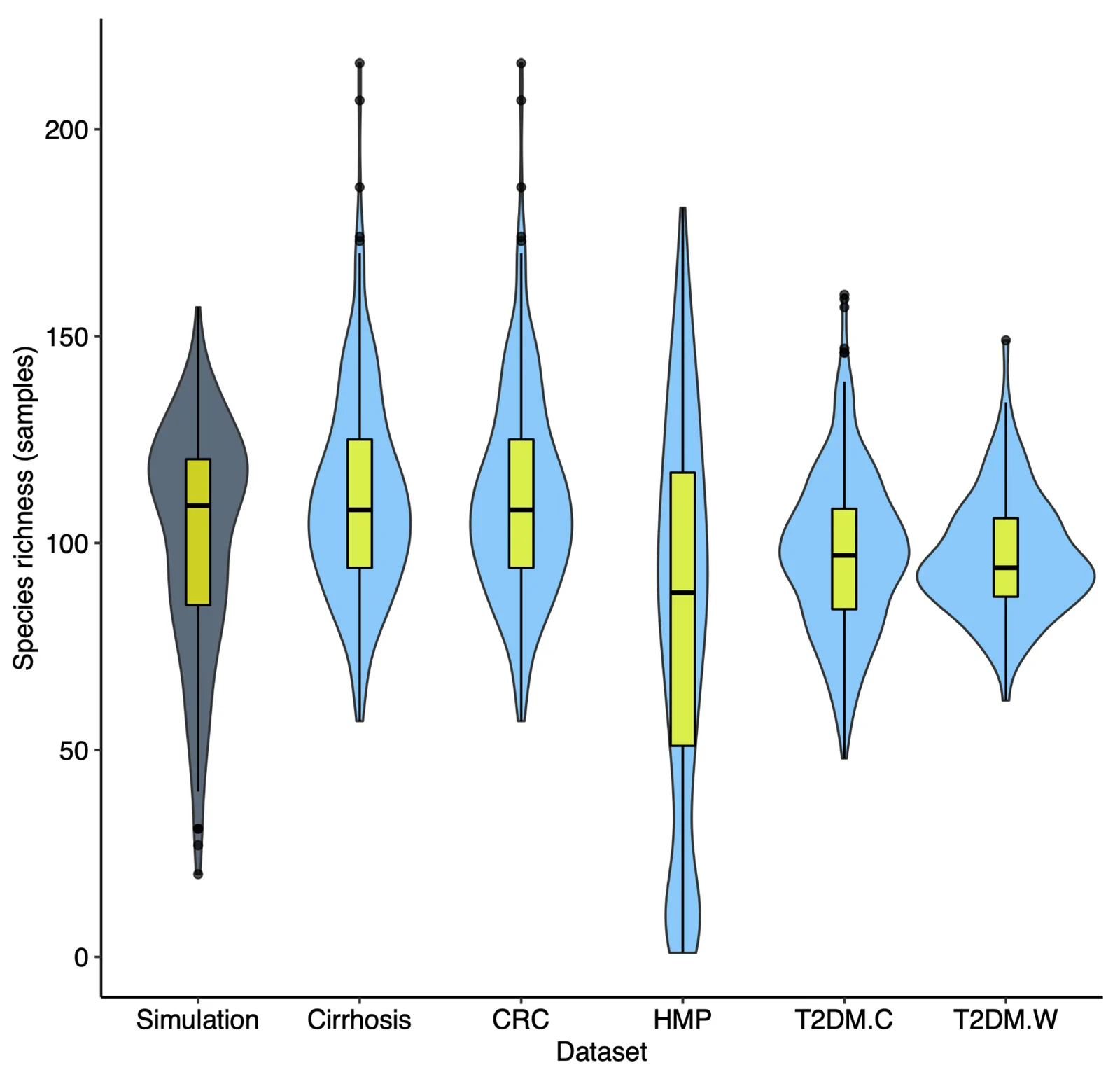
The distribution of α-diversity, i.e., number of species, or species richness, of 250 samples contained in the simulated and real datasets.

**Figure 3:**
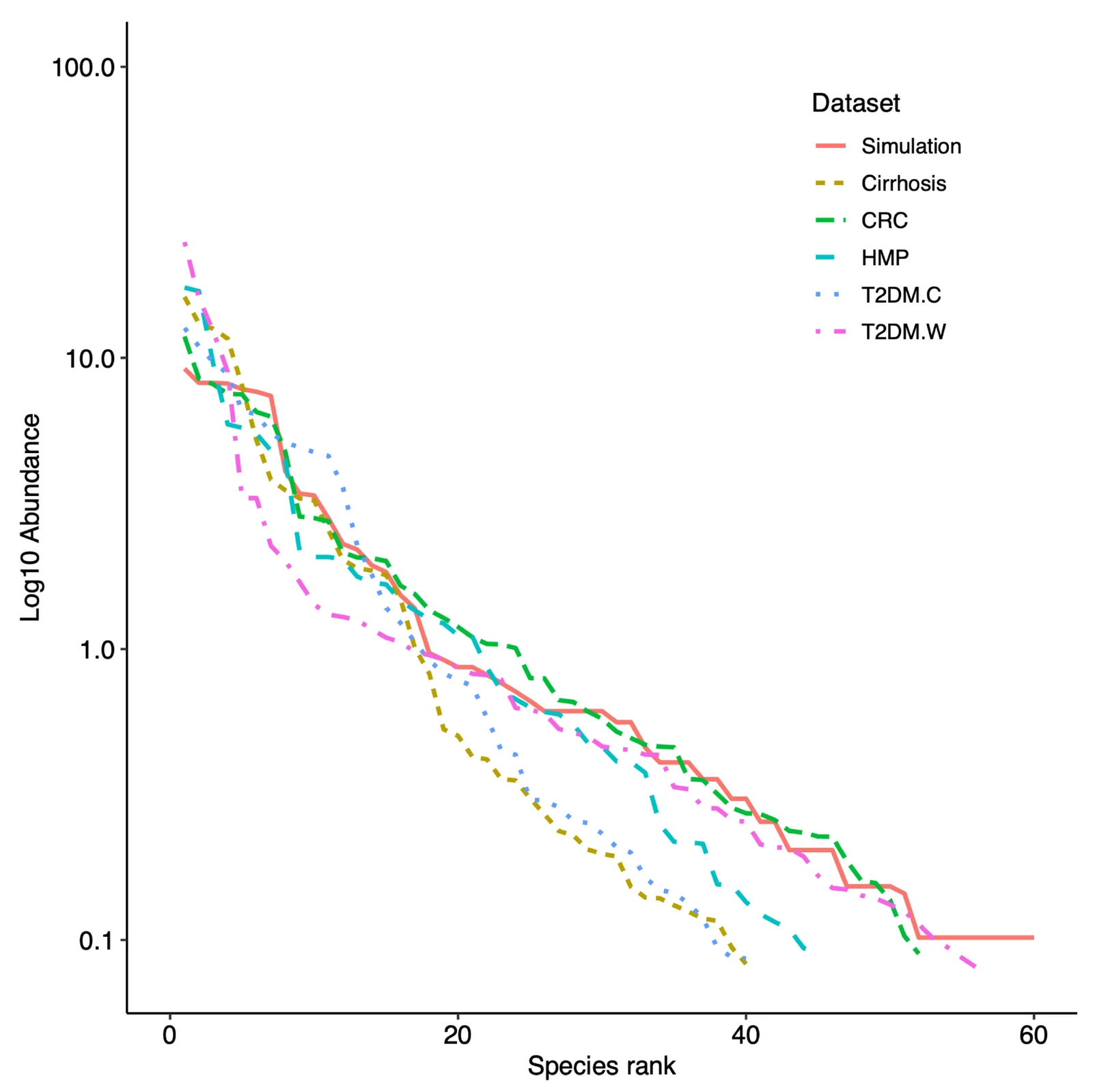
The β-diversity, that is, the number of species represented and how the individuals of 252 microorganisms are distributed among these species.

**Figure 4:**
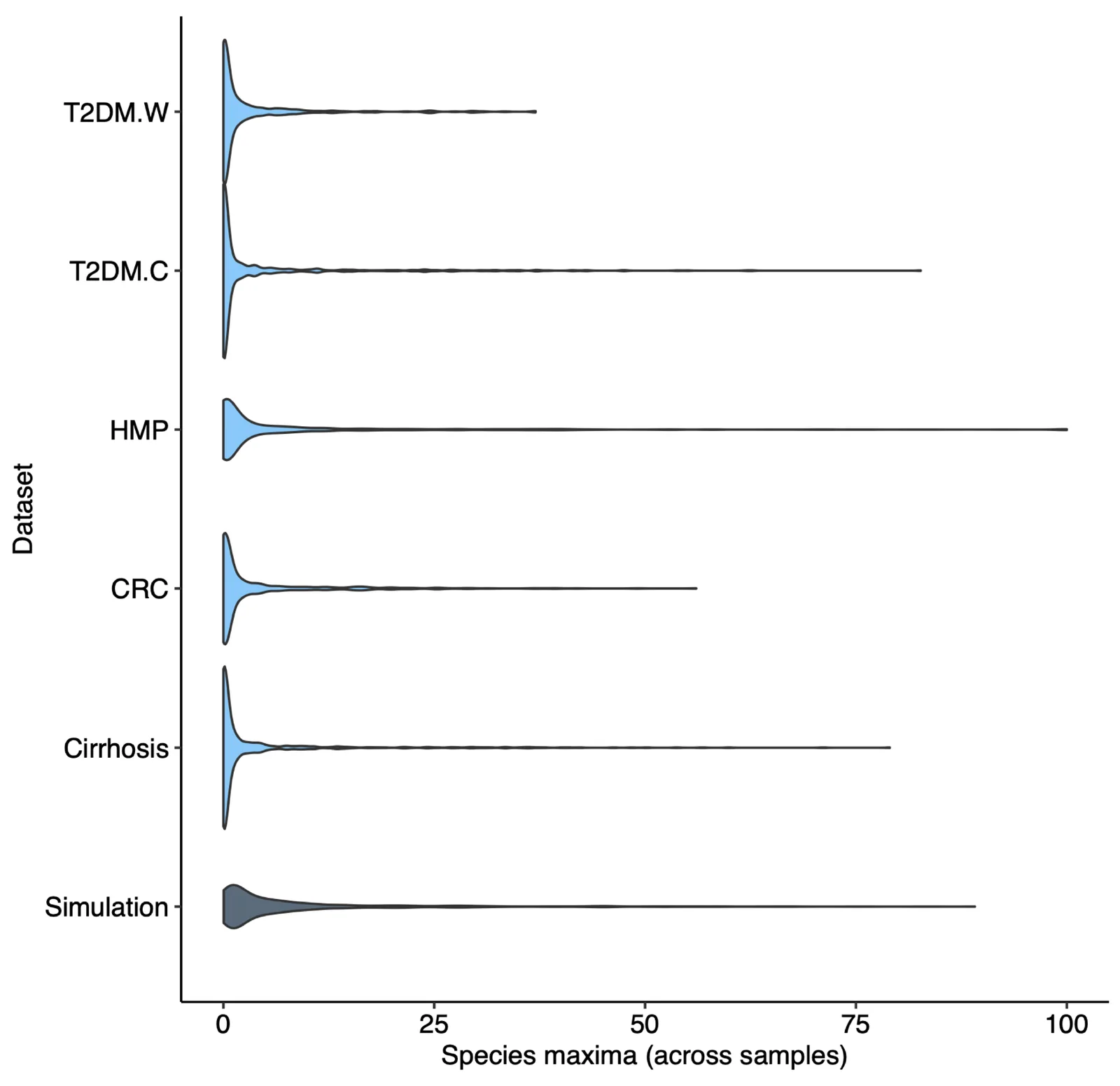
The distribution of each species’ maximum abundance (across all samples) is considered for the six datasets.

**Figure 5:**
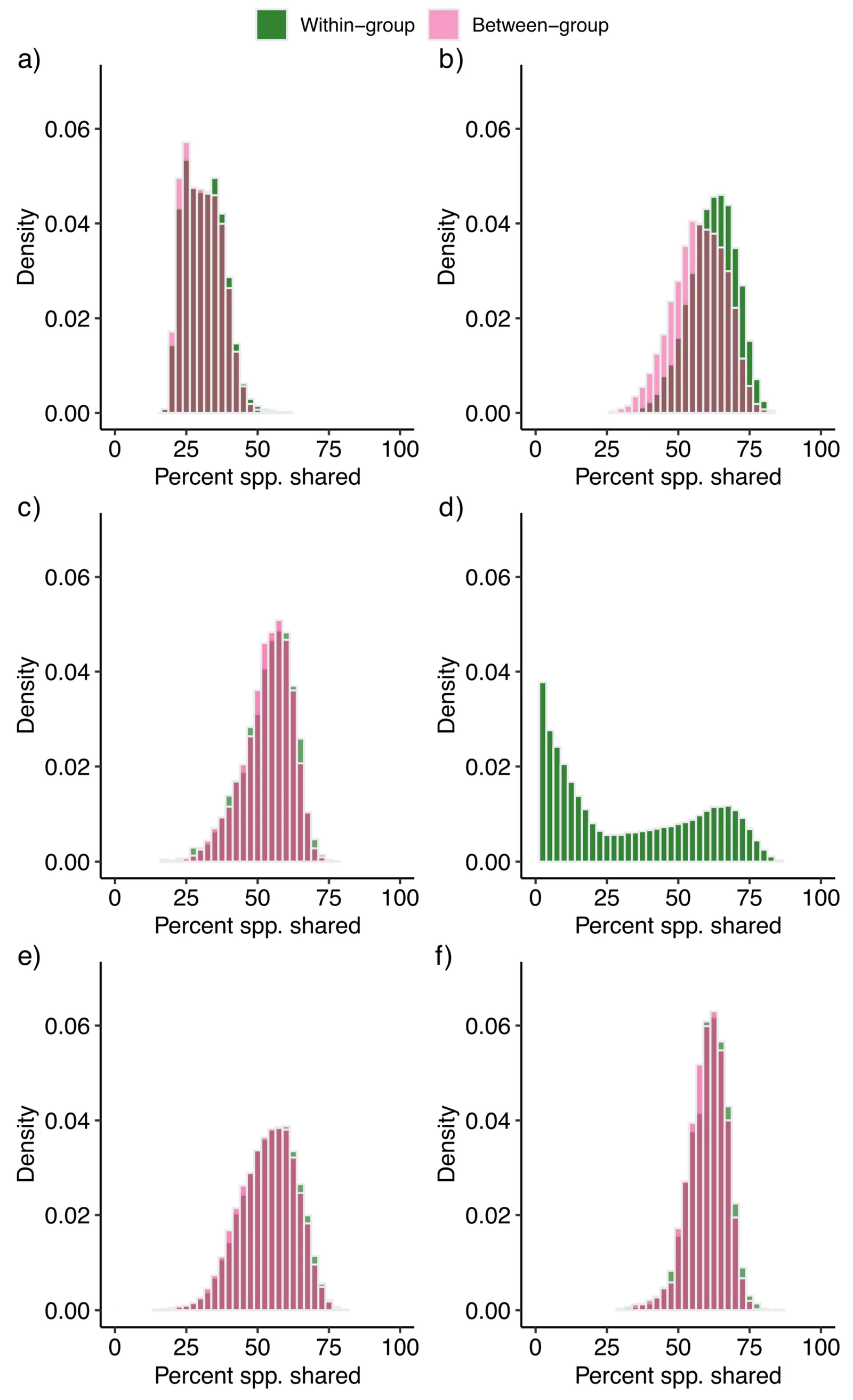
Distribution of the Bray–Curtis index for 278 presence-absence-transformed versions of the (**a**) Synthetic, (**b**) Cirrhosis, (**c**) CRC, (**d**) HMP, (**e**) T2DM.C, and (**f**) T2DM.W datasets.

**Figure 6:**
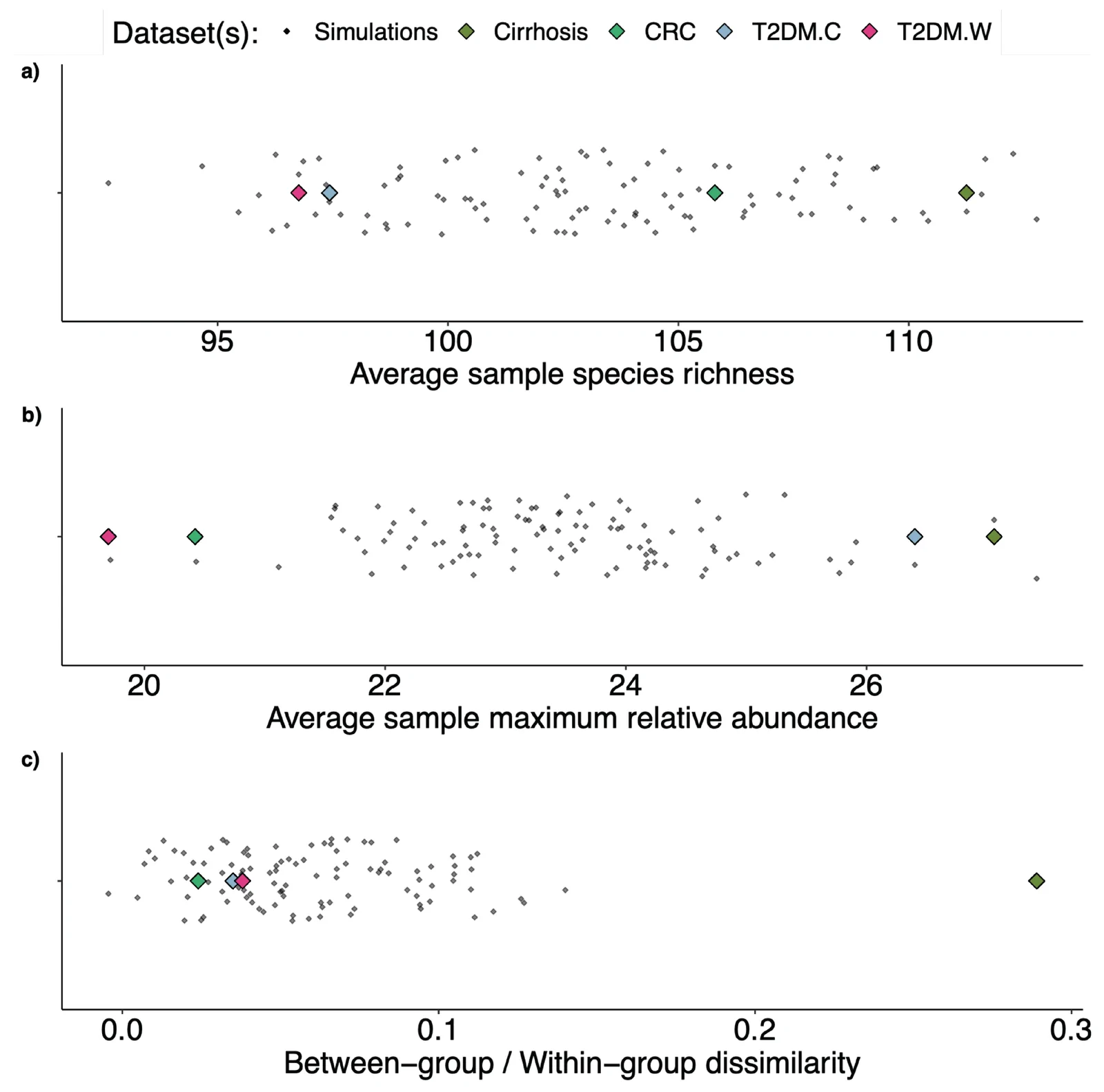
Comparison of four real multi-assemblage datasets with 100 coenocline-simulated datasets for (**a**) average sample species richness, (**b**) average sample maximum relative abundance, and (**c**) between-group rank compositional similarity relative to within-group average rank compositional dissimilarity.

Figure 2, Figure 3, Figure 4, Figure 5 and Figure 6 show how the synthetic and benchmark datasets compare across several types of ecological difference. Figure 2 and Figure 3 demonstrate the species diversity in the benchmark and synthetic data. Figure 2 shows the distribution of α-diversity (i.e., the number of species, or species richness) of samples contained in the synthetic and benchmark datasets. Figure 3, on the other hand, better reflects the β-diversity, that is, the (log_10_-transformed) number of species represented and how individual microorganisms are distributed among these species. The synthetic, Cirrhosis, and CRC datasets show similar levels and distributions of the number of species observed in their samples, although the Cirrhosis and CRC datasets have a small number of samples with very high α-diversity, and the synthetic dataset has a small number of samples with very low species richness. The two Diabetes datasets, while showing a similar average species richness, show less variability in species richness among their respective samples (Figure 2). The HMP dataset shows much more variability in the number of species observed in the samples. However, this is not surprising, given that the HMP data involved samples from several body sites (skin, lung, gut), whereas all four of the other existing datasets were based solely on gut samples.

When we consider the log-rank abundance of randomly selected samples from each dataset (Figure 3), we see quite a lot of resemblance among the six datasets in terms of their diversity. Typically, the samples from each dataset exhibit few very common species and a large (and similar) number of rare species. It should be noted that the abundances in each dataset refer to relative abundances (as in all microbiome datasets), so their values have an upper bound of 100 (%).

In Figure 4, the distribution of each species’ maximum abundance (across all samples) is shown for the six datasets. Again, we see the structure of a few common species and many rare species commonly observed in ecological community datasets. The CRC, Cirrhosis, and Diabetes datasets all show very similar distributions of species maximum abundances. The HMP and synthetic datasets appear to exhibit, to a marginal degree, more intermediate-abundance species compared to the other datasets.

The distributions in Figure 5 represent the Bray–Curtis index for the presence-absence-transformed versions of the synthetic and benchmark datasets. The between- and within-group similarities represent the percentages of species shared among samples from different groups (i.e., cases vs. controls) or the same group (i.e., cases vs. cases or controls vs. controls), respectively. It should be noted that the HMP dataset (Figure 5d) contains a single group (no disease cases), so between-group similarities could not be calculated for this dataset. A perusal of the distributions of sample similarities from each dataset suggests that the synthetic data tend to produce samples with greater compositional dissimilarity; that is, samples from the synthetic dataset typically share fewer species relative to samples from each benchmark dataset (Figure 5b–f). However, when we perused the distributions of the benchmark datasets (Figure 5b–f), we could see that there were major disparities between the distributions associated with each dataset. This is particularly apparent for the HMP dataset, which contained samples that shared almost no species but also contained samples that were almost compositionally equivalent. In terms of the benchmark datasets containing both cases and controls, samples in the Cirrhosis (Figure 5b) and T2DM.W (Figure 5f) datasets were more compositionally similar (medians of approximately 60% of species shared) to those in the CRC (Figure 5c) and T2DM.C (Figure 5e) datasets (medians of approximately 54% of species shared). Of all the case–control datasets we considered, only the Cirrhosis dataset has a visually discernible difference between the distributions of between-group and within-group percentages of species shared among samples. For this dataset, samples belonging to the same group (cases or controls) appear (on average) to share more species (median = 63%) relative to samples belonging to different groups (cases vs. controls; median = 58%). This is not surprising given that, of all the benchmark datasets we considered, the Cirrhosis dataset has the highest level of reported discrimination (accuracy = 87.7% and ROC-AUC = 0.945 based on a random forest classifier).

In Figure 2, Figure 3, Figure 4 and Figure 5, the benchmark datasets were compared with a single, randomly selected synthetic dataset. It is possible that any resemblance between the ecological properties of the synthetic data and those of the existing datasets may have been due to chance. To investigate this, we compared the benchmark datasets with 100 synthetic datasets parameterized in the same way as the single synthetic dataset used in Figure 2, Figure 3, Figure 4 and Figure 5. Figure 6 shows the average number of species observed at each sample within a dataset (Figure 6a), the average sample maximum relative abundance (Figure 6b), and a statistic called ANOSIM-R [27], which compares the average amount of between-group (case vs. control) compositional dissimilarity with the average compositional dissimilarity observed between within-group samples (Figure 6c). Perusal of Figure 6a shows that the synthetic datasets span a similar range of average sample alpha diversity (species richness) to that observed in the benchmark datasets. In terms of the dataset-average sample maximum relative abundance (Figure 6b), the benchmark datasets range from quite low levels of average sample maximum abundance (the T2DM.W and Cirrhosis datasets) of about 20% to quite a high level for the Colorectal Cancer and T2DM.C datasets, which have an average maximum sample relative abundance above 25%. Interestingly, a large majority of the synthetic datasets occupy the range between these two extremes. When we consider how compositionally dissimilar samples from different groups are compared to samples from the same group (Figure 6c), we can see that the Cirrhosis dataset has a considerably larger compositional difference between between-group samples (relative to within-group dissimilarities) and all of the other datasets (existing and synthetic). Again, however, we see that the synthetic datasets appear to yield a feasible range of ANOSIM-R values.

Table 3 shows that the 100 coenocline simulations produced mean sample richness (103.1; range, 92.6–112.8) and ANOSIM-R (0.056; range, 0.00–0.118) values that fell comfortably within the range observed across the four empirical case–control datasets. The mean maximum relative abundance was higher in the simulated dataset (7.31; range, 6.53–8.25) than in the real datasets (2.81–4.03). This feature is readily adjustable: lowering the mean of the modal-abundance parameter $A_{0}$ while keeping the niche breadth constant reduces peak abundances without substantially altering species richness. Overall, the quantitative comparison supports the visual impression that the coenocline framework generates datasets whose key ecological properties are realistic and controllable.

## 4. Discussion

We compared five well-known benchmark microbiome datasets to synthetic case–control data generated using the coenocline simulation method. Specifically, we chose to compare the datasets across common measures used to describe differences between ecological assemblage data: (a) diversity; (b) composition; and (c) abundance. The synthetic datasets we generated appeared to be plausible, capturing ecological properties observed in actual microbiome datasets, and seemed to closely mimic the different types and levels of between-group (case vs. control) differences. We also found that when we generated many datasets (*n* = 100), the variation in ecological properties tended to span those observed across datasets representing different diseases and populations. This suggests that the coenocline approach advocated here is likely to prove useful in interrogating and comparing the relative strengths and weaknesses of statistical- and machine learning-based classification methods used on microbiome samples across a range of research settings (diseases, species, and experimental designs).

One consistent pattern we observed is that samples from the coenocline-simulated datasets tended to share fewer species (lower Bray–Curtis similarity) than samples from most of the empirical case–control datasets. This greater compositional dissimilarity is a direct consequence of the relatively wide niche-breadth and modal-coordinate distributions used in the simulation and can be viewed as a controllable feature rather than a flaw: users can tighten or loosen the species-response parameters to produce stronger or weaker between-group separation. For future classifier benchmarking, this implies that the performance estimates obtained on the current default simulations may represent a somewhat optimistic scenario relative to real datasets that exhibit higher within-group similarity. The framework nevertheless allows the degree of compositional turnover to be tuned systematically, which is precisely the capability needed for thorough methodological evaluation.

Several statistical, machine learning, and ecological tools already exist for simulating microbiome count data. Prominent statistical or template-based examples include SparseDOSSA2 [28], which models taxa with a zero-inflated log-normal distribution linked by a Gaussian copula; MIDASim [29], a fast two-step template-based simulator that preserves both marginal distributions and taxon–taxon correlations; and metaSPARSim [30], a hierarchical model designed primarily for 16S count data. Generative deep learning approaches such as MB-GAN [31] and the more recent DeepBioSim [32] learn latent representations of real abundance tables. Structure-oriented tools such as MCSS [33] focus on generating communities and sequencing reads that reflect the organizational attributes of real microbiomes, while the R/Bioconductor package miaSim (v1.18.0) [34] implements classical ecological models (generalized Lotka–Volterra, neutral models, etc.) for time-series simulation. To our knowledge, the present study is the first to adapt classical coenocline simulation for the generation of synthetic microbiome datasets intended to support the evaluation of classification methods.

These methods excel at reproducing observed sparsity, abundance distributions, correlation structures, or community dynamics and are therefore valuable for method development and benchmarking under realistic statistical or dynamical conditions. However, most remain either data-driven/template-driven or focused on community dynamics rather than on controlled variation along host-associated gradients. Ecological structure of the kind most relevant to disease-status classification (for example, how individual taxa respond to an environmental or disease gradient, niche breadth, or modal location) is either fixed by the chosen template, only indirectly controlled, or not explicitly parameterized. In contrast, the coenocline framework presented here is mechanistically grounded in classical ecological theory. By explicitly defining species-response functions along one or more gradients, it allows users to systematically vary the types and levels of ecological difference (α-diversity, compositional turnover, abundance structure) while still generating realistic count data. This ecological controllability comes at the cost of greater parametric complexity and the need for careful calibration, but it provides a complementary capability that purely statistical or dynamics-oriented simulators currently lack. Computational cost remains modest for the single-gradient settings examined here and scales linearly with the number of taxa and samples. A formal sensitivity analysis examining the relative influence of each coenocline parameter (modal abundance, niche breadth, shape parameters, zero-inflation, and dispersion) on α-diversity, abundance distributions, compositional turnover, and ANOSIM-R was beyond the scope of the present study and remains an important direction for future work.

There are a number of aspects of ecological datasets we did not attempt to emulate in our simulation model, and these limitations should be borne in mind when the simulator is used for future methodological work. First, microbiota may respond to several overlapping gradients (e.g., glucose intolerance and age in patients with diabetes). Although the coenocline framework we employed can specify species-response functions across multiple gradients, we deliberately confined the present simulations to a single disease-severity gradient. This choice matched the structure of the five benchmark case–control datasets [10,11,12,14,24] we used for validation and allowed us to isolate the effects of species-response parameters. Extending the model to multi-gradient settings remains straightforward and is planned for future work. Second, we did not impose additional correlations among species beyond those induced by a shared response to the environmental gradient. Such correlations would represent ecological mechanisms such as competition and symbiosis (i.e., ecological rather than purely physiological species-response functions). We determined that gradient-driven association alone was sufficient for a first demonstration of the approach; explicit interaction networks can be added later if required. Third, the simulations generate absolute abundances that are subsequently converted to relative abundances (or presence/absence) in the same manner as standard microbiome bioinformatic pipelines. Because of the inherent compositionality of the resulting data, closure effects are present. Users who wish to mitigate these effects can apply centered log-ratio (or other compositional) transformations after simulation; the framework itself does not impose any particular transformation. Finally, the five benchmark datasets used for validation (Cirrhosis, CRC, T2DM.C, T2DM.W, and HMP) are well-established and widely reused in the microbiome classification literature, providing a common reference point. They are, however, relatively old, predominantly gut-focused, and each disease is represented by only a single study. The HMP data further differ structurally in that they comprise multi-site samples without a case–control contrast. While these choices maximize comparability with prior benchmarking work, they necessarily limit claims about generalizability to more recent cohorts, other body sites, longitudinal designs, or non-bacterial communities. Expanding the validation set is an important direction for subsequent studies.

We also presented a framework for examining the different ecological properties of microbiome datasets (both real and synthetic data) that better aligns with community ecology. This is something that is lacking in many studies, particularly in those with a more clinical focus, which typically neglect ecological notions of difference such as diversity and composition. To be fair, many of these studies have a very pragmatic objective: to develop models for the purpose of diagnosis, prognosis, or gauging disease progression in specific populations. However, we believe that the information provided in microbiome samples may be very rich and that a more comprehensive exploration is highly desirable, if for no other reason than to gauge the impact of the various types and levels of ecological difference on the ability of classification methods to accurately identify class membership.

In this study, we demonstrated the utility of the coenocline simulation method to generate synthetic microbiome datasets that resemble those seen in the literature. We would like to stress that the purpose here was not to model a particular microbiome assemblage response to a particular disease but rather to produce plausible synthetic microbiome multi-assemblage datasets that can be used for methodological research. Ultimately, the purpose of our study was to present a simulation method that can be used to generate large numbers of synthetic multi-assemblage microbiome datasets that span various design (e.g., sample size, taxonomic resolution, case-to-control ratio) and realistic ecological (e.g., level of compositional difference, level of gradient difference) levels of difference. This would enable assessment of the sensitivity, robustness, and accuracy of statistical and machine learning methods used to analyze microbiome–phenotype associations across the range of diseases and populations encompassed in the area of microbiome research.

## Figures and Tables

**Table 1:** Five shotgun metagenomic benchmark datasets used to assess the validity of the simulation model.

Dataset	# Cases	# Controls	# Species
Cirrhosis [10]	118	114	542
Colorectal Cancer [11]	48	73	507
Human Microbiome Project [24]	NA	981	762
Type 2 Diabetes (Chinese) [12]	170	174	572
Type 2 Diabetes (Women) [14]	53	43	381

All studies included gut samples, except for the Human Microbiome Project (HMP) [24], which comprises samples from multiple body sites and does not include a case–control contrast (NA = not applicable).

**Table 2:** Ecological properties used to compare the real and simulated datasets.

Ecological Property	Within-Dataset Measure	Dataset-Level Measures
Species diversity	Distribution of species richness Log-rank abundance curve	Mean sample species richness
Abundance	Distribution of species’ maxima	Mean sample species maximum abundance
Compositional (dis)similarity	Distribution of species similarity (% of spp. shared)	ANOSIM-R statistic

**Table 3:** Summary of ecological metrics for the five empirical datasets and 100 coenocline simulations.

Dataset(s)	Average Site spp. Richness	Average spp. Max Abundance	ANOSIM-R
Cirrhosis	111.3 (25.2)	4.03 (10.02)	0.153
CRC	105.8 (26.5)	3.72 (7.35)	0.025
T2DM	97.4 (19.5)	3.99 (9.75)	0.035
T2DM(W)	96.8 (14.5)	2.81 (6.02)	0.038
100 simulations *	103.1 [92.6, 112.8]	7.31 [6.53, 8.25]	0.056 [0.0, 0.118]

* Values in square brackets are the minimum and maximum values observed across the 100 independent simulations.

## Data Availability

The authors confirm that the code and simulated data supporting the findings of this study are available within the article and its additional information. They can be found at https://doi.org/10.5281/zenodo.16868084.

## Supplementary Materials

The following supporting information can be downloaded at: https://www.mdpi.com/article/10.3390/pathogens15080877/s1, Table S1. Distributions used for coenocline simulations.
