# Supplementary material for: Coenocline Simulation of Microbiome Samples: A Biologically Mechanistic Framework for Generating Ecologically Realistic Synthetic Datasets to Support Classification Method Evaluation
Source: Pathogens. 2026 Aug 21;15(8):877. doi: 10.3390/pathogens15080877 (PMC13516613; doi:10.3390/pathogens15080877)
Supplement: Supplementary file 1 [file pathogens-15-00877-s001.zip › pathogens-4445816-supplementary.pdf]

Article

Coenocline Simulation of Microbiome Samples: A Biologically Mechanistic Framework for Generating Ecologically Realistic Synthetic Datasets to Support Classification Method Evaluation

Cameron Hurst [1,2,3,4,†], Dhammika Leshan Wannigama [4,5,6,7,8,9,*,†], Eva Malacova [3], Pichaya Tantiyavarong [1], Nop Khongthon [1], Anita Pelecanos [3], Lee Jones [3], Robert Hurst [4] and Gunter Hartel [3,10,*]

1 Department of Clinical Epidemiology, Faculty of Medicine, Thammasat University, Pathum Thani 12120, Thailand; cphurst@gmail.com (C.H.)
2 Center of Excellence in Applied Epidemiology, Thammasat University, Pathum Thani 12120, Thailand
3 Statistics Unit, QIMR Berghofer Medical Research Institute, Brisbane 4006, QLD, Australia
4 Pathogen Hunter's Research Collaborative Team, Department of Infectious Diseases and Infection Control, Yamagata Prefectural Central Hospital, Yamagata 990-2292, Japan
5 Department of Infectious Diseases, Faculty of Medicine, Yamagata University, Yamagata 990-2292, Japan
6 Department of Infectious Diseases and Infection Control, Yamagata Prefectural Central Hospital, Yamagata 990-2292, Japan
7 Biofilms and Antimicrobial Resistance Consortium of ODA Receiving Countries, The University of Sheffield, Sheffield S10 2TN, UK
8 Center of Excellence for Vectors and Vector-Borne Diseases, Faculty of Science, Mahidol University, Bangkok 10400, Thailand
9 Graduate School, Yamagata Prefectural University of Health Sciences, Kamiyanagi, Yamagata 990-2212, Japan
10 Griffith Institute for Biomedicine and Glycomics, Giffith University, Southport 4222, QLD, Australia
* Correspondence: leshanwannigama@gmail.com (D.L.W.); gunter.hartel@qimrb.edu.au (G.H.)
† These authors contributed equally to this work.

Table S1. Distributions used for coenocline simulations.

Component	Parameter	Distribution
**Species response parameters**	**Abundance at optima (A_0)**	$\mathbf{NegBin}(\boldsymbol{\mu}, = \mathbf{4\ size} = \mathbf{0.11})$
	**Optimal abundance coordinate (m)**	$\mathrm{N}(\boldsymbol{\mu}, = \mathbf{50}\ \boldsymbol{\sigma} = \mathbf{12})$
	**Niche breadth (r)**	$\mathbf{Uniform}(\mathbf{30}, \mathbf{80})$
	**Shape parameter α**	$\mathbf{Uniform}(\mathbf{2}, \mathbf{12})$
	**Shape parameter γ**	$\mathbf{Uniform}(\mathbf{2}, \mathbf{12})$
**Host-specific environment (gradient)**	**Control gradient values**	$\mathcal{N}(\boldsymbol{\mu}, = \mathbf{45}\ \boldsymbol{\sigma} = \mathbf{15})$
	**Case gradient values**	$\mathcal{N}(\boldsymbol{\mu}, = \mathbf{55}\ \boldsymbol{\sigma} = \mathbf{15})$
**Count model**	**Observed abundance A_{ij}**	**0 with probability, π = 0.2;** **otherwise** $\mathbf{NegBin}(\boldsymbol{\mu}, = \widehat{A}_{ij}\ \boldsymbol{\theta} = \mathbf{0.125})$

Coenocline simulation was based on the generalized β species-response function (Eqs. 1–3 of the main text). For the i-th species ($i = 1, \ldots, 2000$) and the j-th sample ($j = 1, \ldots, 200$) the expected abundance A_{ij} was calculated and then used as the mean of a zero-inflated negative-binomial distribution with excess-zero probability $\pi = 0.2$ and dispersion $\theta = 0.125$. All parameters were drawn independently.
